# Vaginal microbiota changes of persistent human papillomavirus infection after cervical conization

**DOI:** 10.3389/fcimb.2025.1544794

**Published:** 2025-04-14

**Authors:** Lingyun Liang, Cailing Ma, Yan Li, Yilidana Mijiti, Lipeng Zhang, Yanjia Liu

**Affiliations:** ^1^ The First Affiliated Hospital of Xinjiang Medical University, Department of Gynecology, State Key Laboratory of Pathogenesis, Prevention and Treatment of High Incidence Diseases in Central Asia, Urumqi, China; ^2^ Xinjiang Key Laboratory of Medical Animal Model Research, Urumqi, China

**Keywords:** vaginal microbiota, HPV infection, cervical intraepithelial neoplasia, cervical conization, 16S rDNA

## Abstract

**Objective:**

We investigated the changes in vaginal microbiota among females with persistent human papillomavirus (HPV) infection following cervical conization in Xinjiang, China.

**Methods:**

A total of 108 female participants were enrolled in the study, including 37 HPV-positive females without cervical conization (Group P1), 37 HPV-positive females after cervical conization (Group P2), and 34 HPV-negative females after cervical conization (Group N). DNA was extracted from vaginal secretions, and the V3-V4 regions of bacterial 16S rDNA were amplified and sequenced using NovaSeq technology. The diversity analysis of the bacterial microbiota was conducted using QIIME2 and R software, while the phenotypic analysis was performed with Bugbase software.

**Results:**

Lactobacillus was the predominant genus in the vaginal microbiota of women with persistent HPV infection after cervical conization in Xinjiang. Following partial cervical resection, the α-diversity of the vaginal microbiota decreased, particularly among patients who had cleared HPV. Bacterial vaginosis-associated anaerobes were common in the vaginal environment, with their relative abundance increasing in cases of persistent HPV infection. Postoperative persistent HPV infection was found to be correlated not only with pathogens linked to bacterial vaginosis but also with those associated with aerobic vaginitis. Gardnerella and Atopobium, as well as Bifidobacterium and Streptococcus, demonstrated a symbiotic synergy. Both Lactobacillus and Gardnerella exhibited negative correlations with many pathogenic bacteria. Anaerobic and biofilm formation were the most evident phenotypes in individuals with persistent HPV infection after conization.

**Conclusion:**

The vaginal microbiota of women with persistent HPV infection following cervical conization is characterized by the coexistence of Lactobacillus dominance and increased microbial diversity. Anaerobic bacteria and biofilm formation may play a significant role in the persistence of HPV infection post-surgery, and the role of Gardnerella in the vaginal flora under an HPV-infected state warrants further study.

## 1 Introduction

Persistent high-risk human papillomavirus (HR-HPV) infection is closely associated with the development of cervical squamous intraepithelial lesions and cervical cancer (D’Augè et al., 2024). Conization of the cervix is a critical procedure for treating high-grade squamous intraepithelial lesion (HSIL), however, some patients experience persistent HPV infection following partial cervical resection. The risk of recurrent cervical lesions is significantly higher compared to that in the general population (van der Heijden et al., 2015), thus, they constitute significant targets for clinical follow-up. Currently, there is no effective treatment for persistent HPV infection post-surgery, and many patients are over 45 years old, significantly reducing the efficacy of the HPV vaccine’s immune defense in this age group (Castle et al., 2009). As research on vaginal microbiota (VMB) gains popularity, an increasing number of scholars are discovering that disruptions in VMB may facilitate the occurrence or persistence of HPV infection (Sharifian et al., 2023), but restoring the VMB balance can facilitate the reversal of HPV infection (Palma et al., 2018; Liu et al., 2024). Research has indicated that the presence of anaerobic bacteria tends to rise in the vaginal environment of those with HPV infection (Xia et al., 2022), as well as HPV-associated cervical lesions or cervical cancer (Wu et al., 2021; Bai et al., 2024). Metabolites secreted by these bacteria play a role in certain pathways (Uren et al., 2005; Huang et al., 2024). There are few studies on the vaginal microbiota of persistent HPV infection following cervical conization. This study aims to investigate whether there are differences in the microbiota structure between cases with and without persistent HPV infection following conization, as well as to determine if there are structural variations in the microbiota between persistent HPV infection post-surgery and pre-surgery. These questions will be the central focus of our research.

## 2 Materials and methods

### 2.1 Study participants

Female patients treated in the gynecological department of the First Affiliated Hospital of Xinjiang Medical University from March 2024 to July 2024 were recruited. Inclusion criteria were as follows: (1) Ages ranging from 25 to 60 years old. (2) Sexual history of one year or more. (3) At least two HPV tests had been reported in this hospital, with an interval of six months or more. (4) Cervical biopsy or cervical conization procedure had been conducted in this hospital. Exclusion criteria were as follows: (1) In period of pregnancy or menstruation. (2) When obtaining vaginal secretions, the presence of vaginal bleeding or obvious bloody discharge. (3) Sexual activity within one week prior to collection. (4) A history of vaginal douching or medication use within three months preceding collection. (5) Use of antibiotics or sex hormones within three months prior to collection. (6) Past history of hysterectomy, radiotherapy or chemotherapy for genital tract tumors. (7) Other malignant tumor conditions or systemic immune disorders.

### 2.2 Grouping standards

Group P1: Preoperative persistent HPV-positive group. Patients diagnosed with HR-HPV infection for a duration of 6 months or more, were confirmed by at least two separate HPV typing tests conducted in our hospital. Simultaneously, thin-layer liquid-based cytology testing (TCT) was conducted, with or without subsequent colposcopy examination or biopsy. Group P2: Postoperative persistent HPV-positive group. Patients underwent cervical conization due to HSIL and had a history of HR-HPV infection prior to surgery.Following the surgery, they underwent at least two subsequent HPV typing tests that confirmed the persistence of the HPV infection. TCT was completed simultaneously during the follow-up, with or without subsequent colposcopy examination or biopsy. Group N: Postoperative persistent HPV-negative group. Patients underwent cervical conization due to HSIL, with a history of HR-HPV infection prior to surgery. Following the surgery, they underwent at least two subsequent HPV typing tests that confirmed the clearance of the HPV infection. Concurrently, TCT was performed during the follow-up, with negative results.

### 2.3 16S rDNA sequencing

We used sterile speculums to expose the vagina and cervix, and then collected vaginal secretions from the vaginal fornix using sterile cotton swabs. The swabs were stored at −80°C in 15 minutes. Genomic DNA was extracted from the swabs according to the Universal Genomic DNA Kit (CW2298M, Kangwei Century Biotechnology Co., Jiangsu, China). DNA concentration was quantified using Nano-Drop 2000. The primers 341F (5′-CCTACGGGNGGCWGCAG-3′) and 805R (5′-GACTACHVGGGTATCTAATCC-3′) were used to amplify V3–V4 hypervariable regions of the bacterial 16S rDNA. The PCR amplification system (25μL) included 12.5μL Pusion Hot Start Flex 2X Master Mix (M0536L, Yitao Biological Instrument Co., Shanghai, China), 2.5μL upstream primer, 2.5μL downstream primer and 50ng DNA template. The PCR amplification procedure was conducted as follows: predenaturation at 98°C for 30s; 35 cycles including denaturation at 98°C for 10s, annealing at 54°C for 30s, and extension at 72°C for 45s; additional extension at 72°C for the last 10mins. Subsequently, the PCR products were confirmed using 2% agarose gel electrophoresis and purified with AMPure XT beads (Beckman Coulter Genomics, Danvers, MA, USA). The concentration was determined by the Qubit fluorometer (Invitrogen, USA). The size and quantity of the amplicon libraries were evaluated on the Agilent 2100 Bioanalyzer (Agilent, USA) and the Library Quantification Kit for Illumina (Kapa Biosciences, Woburn, MA, USA), respectively. Finally, DNA libraries with a concentration above 2nM were deemed qualified and sequenced by LC-Bio Technology Co., Ltd. (Hangzhou, China) using the NovaSeq 6000 platform.

### 2.4 Sequencing data processing and analysis

QIIME2 (Quantitative Insights into Microbial Ecology) was utilized to analyze the sequencing data. After splicing overlapping sequences, conducting quality control, and filtering chimeras from the raw data, high-quality and clean data was obtained. Following the dereplication process using DADA2 (Divisive Amplicon Denoising Algorithm 2), amplicon sequence variant (ASV) feature tables and their corresponding feature sequences were obtained. Taxonomic identification of ASVs was performed using the Silva (Release 138) database. α-diversity and β-diversity were calculated with R software (version 3.4.4). Differences in species abundance among the three groups were detected using Kruskal–Wallis and Wilcoxon rank sum tests. The Linear discriminant analysis effect size (LEfSe) online tool (https://www.omstudio.cn/tool/) was used to analyze the differential bacteria among the three groups. Species phenotype was analyzed using Bugbase software (https://bugbase.cs.umn.edu/).

### 2.5 Statistical analysis

The analysis was conducted using SPSS 23.0 software. The normality of the measurement data was assessed through the Kolmogorov–Smirnov test. Due to the non-normal distribution of the data, it was represented using the median and interquartile range. To compare differences among groups, the Kruskal-Wallis rank sum test was employed. Enumeration data was expressed as percentages or rates (%), and analyzed with the chi-square test. Pearson’s correlation coefficient was used for species correlation analysis. *P* < 0.05 was statistically significant.

## 3 Results

### 3.1 Basic characteristics of participants

A total of 120 patients were enrolled in this study; however, 12 individuals were excluded due to deviant samples. Of the remaining 108 participants, 37 cases were in the preoperative persistent HPV-positive group (Group P1), 37 cases were in the postoperative persistent HPV-positive group (Group P2), and 34 cases were in the postoperative persistent HPV-negative group (Group N). No significant differences were observed among the groups in terms of age, ethnicity, menopausal status, number of pregnancies, number of deliveries, timing of vaginal secretions collection, HPV infection status, cervical biopsy results, lesion grades from cervical conization, and margin status of cervical conization.(*P* > 0.05, **Table 1**).

**Table 1:** Basic characteristics of participants.

	Total	Group P1	Group P2	Group N	*P* value
No. of cases (n)	108	37	37	34	
Age (years), median (range)	45,0 (37,0-53,0)	46,0 (37,0-53,0)	48,0 (37,0-53,0)	43,0 (37,8-49,0)	0,306^a^
Ethnicity, n (%)					0,948^b^
Han	92 (85,2%)	31 (83,8%)	32 (86,5%)	29 (85,3%)	
others	16 (14,8%)	6 (16,2%)	5 (13,5%)	5 (14,7%)	
Menopause status, n (%)					0,267^b^
Yes	44 (40,7%)	17 (45,9%)	17 (45,9%)	10 (29,4%)	
No	64 (59,3%)	20 (54,1%)	20 (54,1%)	24 (70,6%)	
Number of pregnancies (n)	2,0 (1,0-3,0)	2,0 (1,0-3,0)	3,0 (2,0-4,0)	2,0 (1,0-3,3)	0,495^a^
Number of deliveries (n)	1,0 (1,0-2,0)	1,0 (1,0-2,0)	1,0 (1,0-2,0)	1,0 (1,0-2,0)	0,867^a^
Timing of vaginal secretions collection ^c^, n (%)					0,690^b^
HPV infection>1 year	54 (50,0%)	17 (45,9%)	18 (48,6%)	19 (55,9%)	
HPV infection ≤ 1 year	54 (50,0%)	20 (54,1%)	19 (51,4%)	15 (44,1%)	
HPV infection status 1^d^, n (%)					0,338^b^
HPV16/18	28 (37,8%)	12 (32,4%)	16 (43,2%)	–	
other 12 types of HPV	46 (62,2%)	25 (67,6%)	21 (56,8%)	–	
HPV infection status 2^d^, n (%)					0,572^b^
mixed HPV types	16 (21,6%)	7 (18,9%)	9 (24,3%)	–	
single HPV type	58 (78,4%)	30 (81,1%)	28 (75,7%)	–	
Cervical biopsy results ^d^, n (%)					0,963^b^
HSIL	12 (16,2%)	6 (16,2%)	6 (16,2%)	–	
LSIL	19 (25,7%)	10 (27,0%)	9 (24,3%)	–	
cervicitis	43 (58,1%)	21 (56,8%)	22 (59,5%)	–	
Lesion grades from cervical conization ^e^, n (%)					0,511^b^
HSIL/CIN2	41 (57,7%)	–	20 (54,1%)	21 (61,8%)	
HSIL/CIN3	30 (42,3%)	–	17 (45,9%)	13 (38,2%)	
Margin status of cervical conization ^e^, n (%)					0,931^b^
positive	4 (5,6%)	–	2 (5,4%)	2 (5,9%)	
negative	67 (94,4%)	–	35 (94,6%)	32 (94,1%)	

(a) Kruskal-Wallis rank sum test with median (1/4-3/4 interquartile range). (b) Chi-square test with n%. (c) Group P1 begins with the first discovery of HPV infection, Groups P2 and N begin with cervical conization. (d)The current infection status, only including Groups P1 and P2. HSIL is high-grade squamous intraepithelial lesion, LSIL is low-grade squamous intraepithelial lesion. (e)With history of cervical conization, only including Groups P2 and N. HSIL is divided into cervical intraepithelial neoplasia (CIN) 2 and 3.

### 3.2 Sequencing results

**Figure 1:**
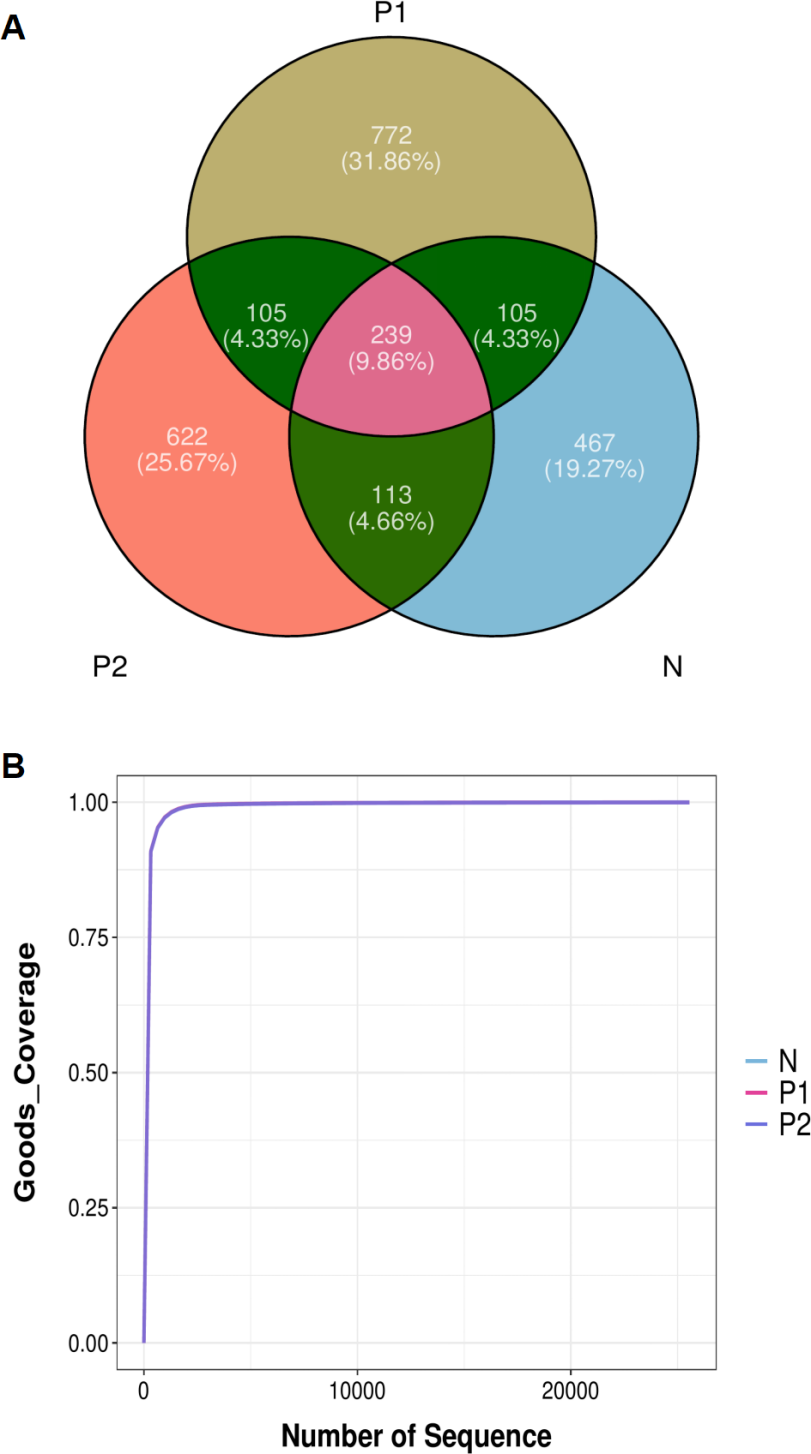
Abundance of vaginal microbiota. **(A)** Venn diagram of ASVs. **(B)** Sequencing dilution curves of samples.

We obtained 6,571,622 clean reads from 108 samples, with an average of 60,848 ± 8,668 reads per sample. A total of 2,423 ASVs were obtained, with 239 ASVs shared among the three groups. Group P1 had the richest ASVs, while Group N had the least ASVs (**Figure 1A**). Upon annotation, a total of 31 phyla, 276 families, 594 genera, and 818 species were identified. The overlapping sequencing dilution curves of the samples in the three groups indicated that the sequencing depths were appropriate (**Figure 1B**).

### 3.3 Diversity of vaginal microbiota

**Figure 2:**
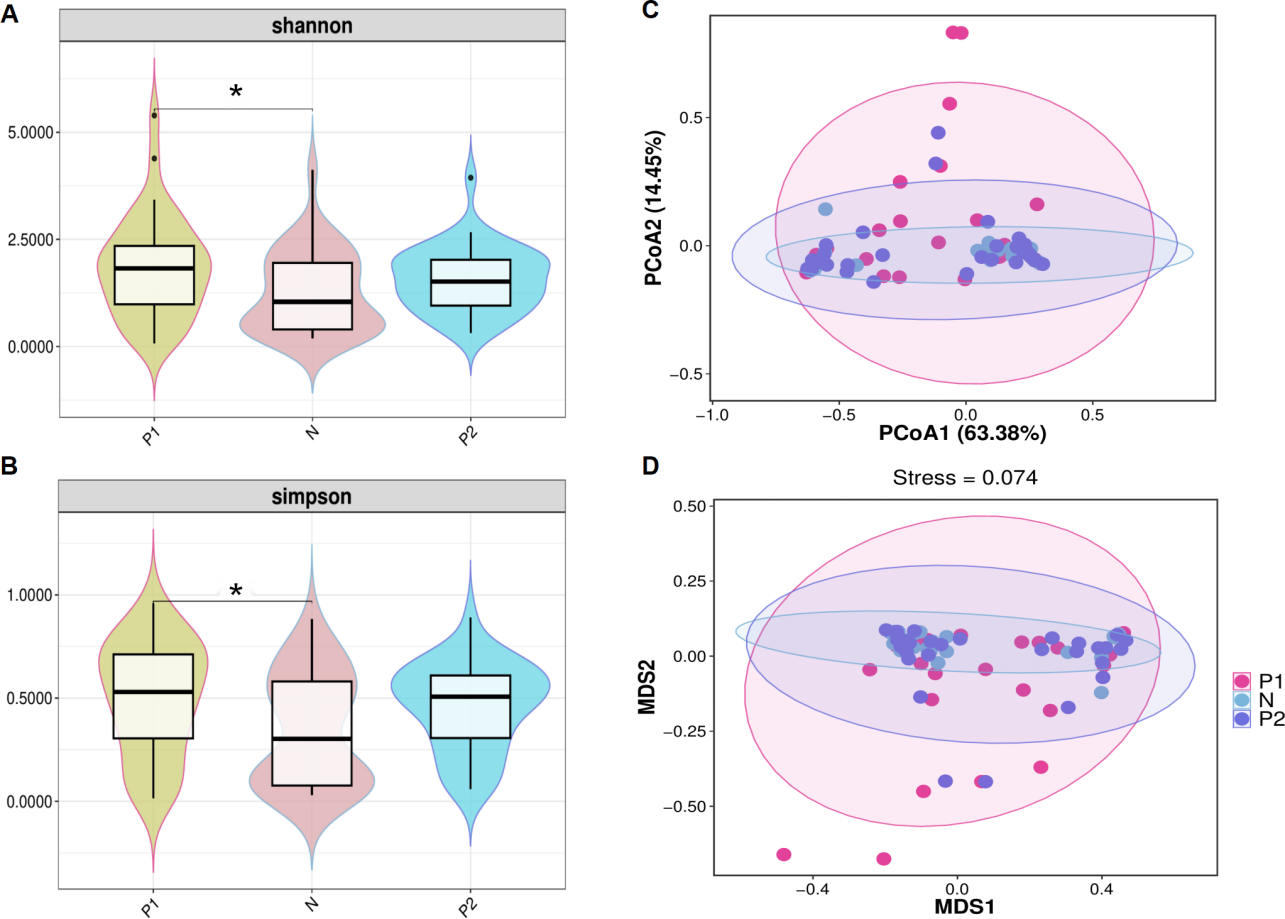
α-diversity and β-diversity of the vaginal microbiota. **(A)** Violin plot of shannon index among three groups. **(B)** Violin plot of simpson index among three groups. For **(A, B)**, the dark horizontal bar represents the median value of each group, while the boxes represent the 25th and 75th percentile values. * *P <*0.05. **(C)** PCoA analysis based on the weight-UniFrac distance. **(D)** NMDS analysis based on the weight-UniFrac distance. For **(C, D)**, the percentages of the abscissa and ordinate represent the interpretation of the sample gap by this dimension.

The α-diversity of vaginal microbiota (VMB) was evaluated using the Shannon and Simpson indices. Both indices comprehensively assess the abundance and evenness of species. The Shannon index for Group P1 (1.84 ± 1.18) was significantly higher than that of Group N (1.27 ± 0.96, *P* < 0.05). No significant difference was observed between Group P2 (1.57 ± 0.76) and Group N, nor between Group P1 and Group P2, *P* < 0.05 (**Figure 2A**). Similarly, the Simpson index for Group P1 (0.50 ± 0.27) was significantly higher than that of Group N (0.35 ± 0.27, *P* < 0.05). Additionally, there was no significant difference between Group P2 (0.46 ± 0.21) and Group N, nor between Group P1 and Group P2, with *P* < 0.05 (**Figure 2B**).

### 3.4 Structure of vaginal microbiota

Principal Coordinates Analysis (PCoA) and Nonmetric Multidimensional Scaling (NMDS) analyses were conducted to evaluate β-diversity among the three groups. In the PCoA, the two principal components accounted for 63.38% and 14.45% of the variance, respectively (**Figure 2C**). In the NMDS, the stress value was less than 0.1 (**Figure 2D**). However, Group N largely overlapped with Group P2, and both Group N and Group P2 exhibited considerable overlap with Group P1, suggesting that the microbiota of the three groups are similar.

**Figure 3:**
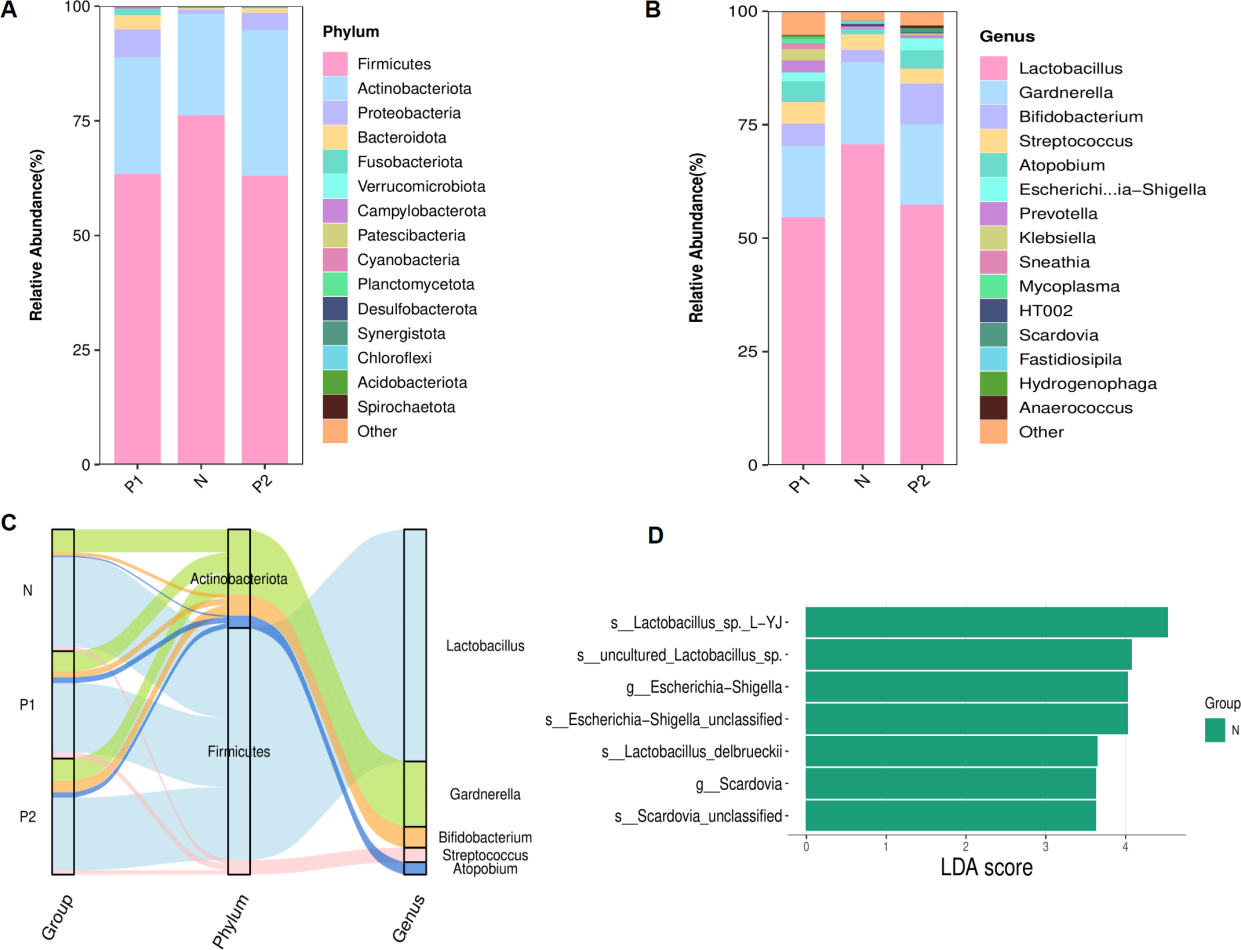
Structure of the vaginal microbiota. **(A)** Stacked bar chart of relative abundances for TOP 15 richest phyla. **(B)** Stacked bar chart of relative abundances for TOP 15 richest genera. **(C)** Sankey plot of the TOP 5 richest genera, corresponding to the affiliated phyla. **(D)** Histogram showed the LEfSe analysis for signifcantly diferential bacteria from genus to species level (LDA score ≥ 3.0).

The analysis of vaginal microbiota structure was conducted based on the relative abundances of bacteria. At the phylum level, the three groups consistently exhibited the top five phyla: Firmicutes, Actinobacteriota, Proteobacteria, Bacteroidota, and Fusobacteriota (**Figure 3A**). Despite variations in the relative abundances of these top five phyla across the groups, no statistically significant differences were observed (**Table 2-1**, *P* > 0.05).

**Table 2-1:** Relative abundances of the top five phyla.

Groups	Firmicutes	Actinobacteriota	Proteobacteria	Bacteroidota	Fusobacteriota
Group P1 (%)	63,6	25,5	6,1	3,2	1,2
Group P2 (%)	63,2	31,9	3,7	1,0	0,1
Group N (%)	76,5	22,2	0,8	0,4	0,2

**Table 2-2:** Relative abundances of the top five genera.

Groups	Lactobacillus	Gardnerella	Bifidobacterium	Streptococcus	Atopobium
Group P1 (%)	54,8	15,8	4,9	4,8	4,5
Group P2 (%)	57,6	17,8	9,0	3,2	4,1
Group N (%)	70,9	18,1	2,7	3,4	1,2

At the genus level, the top five genera consistently observed across the three groups were Lactobacillus, Gardnerella, Bifidobacterium, Streptococcus and Atopobium (**Figure 3B**), each with a relative abundance exceeding 1.0%. The top five genera and their phylum-level affiliations are depicted in **Figure 3C**. Lactobacillus was identified as the predominant genus across the three groups. No statistically significant differences were observed among the top five genera within each of the three groups (refer to **Table 2-2**, *P* > 0.05). However, the relative abundance of Escherichia-Shigella in Group P2 was markedly elevated compared to Group N (2.6% vs 0.1%, *P* = 0.01), as was Scardovia in Group P2 compared to both Group P1 and Group N (0.8% vs 0.0% vs 0.0%, *P* = 0.01). LEfSe analysis further confirmed that Escherichia-Shigella and Scardovia were signature microbes in group N at the genus level (*P* < 0.05, **Figure 3D**).

At the species level, due to the limitations of 16S rDNA amplicon sequencing technology, only about 50.0% of bacteria could be accurately classified (47.0% in Group P1, 32.8% in Group P2, and 50.5% in Group N). Among the annotated species, Lactobacillus crispatus was the dominant bacterium with no significant difference among the three groups (21.1% in Group P1, 13.2% in Group P2, and 29.2% in Group N, *P* > 0.05).

### 3.5 Correlation analysis of vaginal microbiota

Correlation analysis was conducted at the genus level to explore associations among vaginal microbes, focusing on the relative abundances of the top ten genera across three groups. Filtration conditions were set as follows: correlation coefficient r > 0.5 or r < -0.5, *P* < 0.05. Bifidobacterium and Streptococcus exhibited strong positive correlations, with r = 0.80 in Group P1, r = 0.82 in Group P2, and r = 0.99 in Group N, *P* < 0.001. Similarly, Atopobium and Gardnerella also demonstrated positive correlations, with r = 0.69 in Group P1, r = 0.75 in Group P2, and r = 0.86 in Group N, *P* < 0.001.

**Figure 4:**
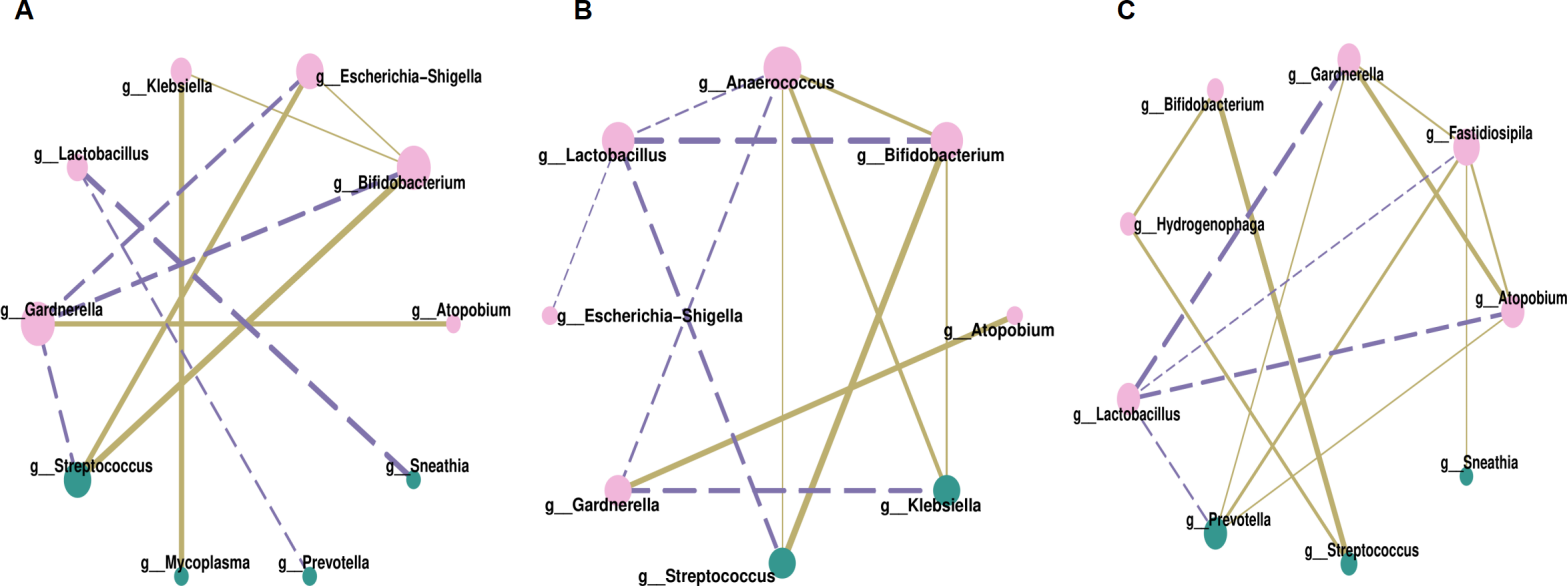
Correlation network analysis of vaginal microbiota, **(A)** for Group P1, **(B)** for Group P2, **(C)** for Group N. Each node in the figures represented a dominant genus, larger node and darker color indicated more related objects of the bacterium. The line between two nodes showed they were related, thicker line indicated stronger correlation. Solid line represented positive correlation while dotted line represented negative correlation.

The bacteria negatively associated with Lactobacillus were not consistent across the three groups. In Group P1 (**Figure 4A**), they were Sneathia (r = -0.75) and Prevotella (r = -0.59), *P* < 0.001. In Group P2 (**Figure 4B**), they were mainly Bifidobacterium (r = -0.79) and Streptococcus (r = -0.68), *P* < 0.001. And in Group N (**Figure 4C**), they were Gardnerella (r = -0.88), Atopobium (r = -0.78), Prevotella (r = -0.65), and Fastidiosipila (r = -0.58), *P* < 0.001. However, the negative correlations became more complex within the HPV infection groups. In addition to negative correlations with Lactobacillus, there were also negative correlations with Gardnerella, such as Bifidobacterium (r = -0.69), Streptococcus (r = -0.66), Escherichia-Shigella (r = -0.66) in Group P1, as well as Klebsiella (r = -0.71), Anaerococcus (r = -0.59) in Group P2, *P* < 0.001.

### 3.6 Phenotype analysis of vaginal microbiota

**Figure 5:**
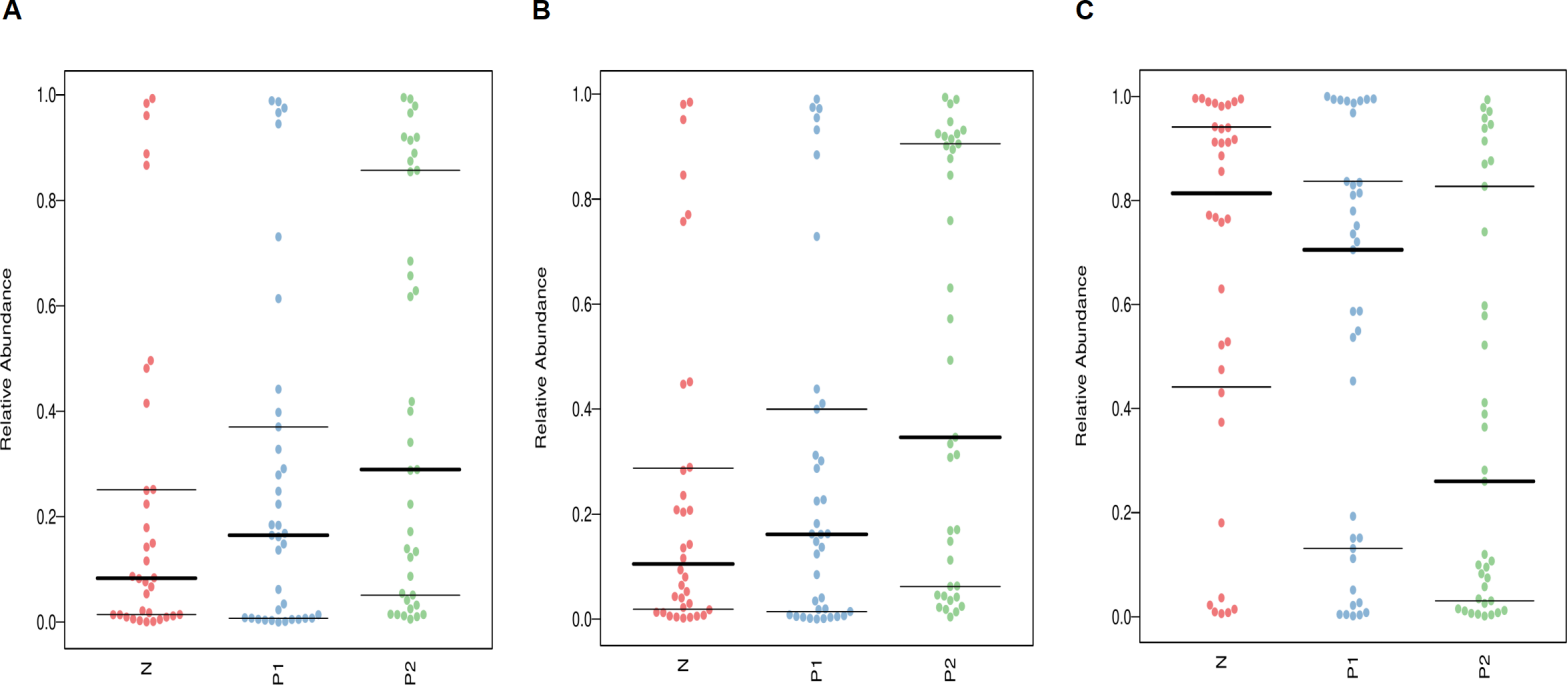
Phenotype analysis of vaginal microbiota, **(A)** for Anaerobic, **(B)** for Forms Biofilms, **(C)** for Facultatively Anaerobic. The thick horizontal line represents the median value of each group, while the thin horizontal lines represent the 25th and 75th percentile values, *P* < 0.05.

Understanding the phenotypes of vaginal microbes is crucial for clinical interventions. The microbial phenotype analysis was conducted using BugBase software. As depicted in **Figure 5**, Group P2 exhibited the highest relative abundances in terms of Anaerobic (**Figure 5A**) and Forms Biofilms (**Figure 5B**), but the lowest in Facultatively Anaerobic (**Figure 5C**) among the three groups, *P* < 0.05.

## 4 Discussion

In this study, we compared the vaginal microbiota (VMB) between females with and without cervical conization, aiming to provide support for the management of postoperative patients. This marks the first exploration of the characteristics of the VMB in females with persistent HPV infection following cervical conization, utilizing 16S rDNA amplicon sequencing technology in Xinjiang, China.

The VMB of Asian females in reproductive age has been reported Lactobacilli-dominated (Ravel et al., 2011). Zeng et al. (2023) analyzed the vaginal flora of patients aged 18 to 60 in Shantou, China, finding that the dominant bacterium was Lactobacillus in both HPV-infected patients (>43.0%) and control individuals (39.1%). Chen et al. (2020) evaluated the association between VMB and HPV infection, as well as HPV-related squamous intraepithelial lesions in Shanghai, China. They found that Lactobacillus was the most abundant genus (>42.0%) across all groups aged 25 to 69 years old. In this study, we obtained similar results, with the relative abundances of Lactobacillus being 70.9% in the postoperative persistent HPV-negative group, 57.6% in the postoperative persistent HPV-positive group, and 54.8% in the preoperative persistent HPV-positive group. Each of these three studies involved postmenopausal females as subjects.

Simultaneously, some anaerobic bacteria were also commonly present in the vaginal environment (Fang et al., 2022). In our analysis of VMB structure, the main anaerobic genera were Gardnerella, Bifidobacterium, Streptococcus and Atopobium. It was quite different to the report of Mi Zeng et al (Zeng et al., 2023), with Gardnerella, Sneathia, Prevotella and Klebsiella as dominant anaerobes, but similar to another study (Xia et al., 2022) in Xinjiang, China, with Gardnerella, Atopobium, Bifidobacterium and Sneathia as dominant anaerobes. This may be related to population or regional differences.

However, the relative abundances of the top five genera varied among the three groups, showing a decreasing trend of Lactobacillus and an increasing trend of bacterial vaginosis (BV)-associated anaerobes from the HPV-negative group to the HPV-positive groups, with the exception of Gardnerella. Correspondingly, the α-diversity of the VMB in the preoperative HPV-positive group was significantly higher than that in the postoperative HPV-negative group, with the postoperative HPV-positive group exhibiting an intermediate state. Early in 2013, Gao et al. (2013) detected that diversity of VMB in the HPV-positive group was higher than that in the HPV-negative group. However, the detailed molecular mechanism has yet to be fully understood. Lactobacillus may play a crucial role in maintaining the cervical epithelial barrier function, thereby hindering the entry of HPV into basal keratinocytes through the production of low pH and bacteriocin (Borgdorff et al., 2015). Metabolites also differ between individuals who are HPV-positive and those who are HPV-negative. For instance, there is an increase in biogenic amines, but a decrease in glutathione in cases of HPV infection (Borgogna et al., 2020).

The impact of cervical conization on vaginal flora was observed to be consistent with findings from other research. A prospective study (Caselli et al., 2020) compared the VMB of 85 high-grade squamous intraepithelial lesions (HSILs) before and after conization, finding that surgical removal of HPV-associated cervical lesions induced microbiome reconstruction. This resulted in a significant decrease of community state type (CST)-IV in high-risk HPV-cleared patients, compared to an unaltered prevalence in high-risk HPV-persistent patients. Ravel et al. (2011) indicated that CST-IV was characterized by higher proportions of strictly anaerobic bacteria. Li et al. (2022) investigated the correlation between vaginal microbiota dysbiosis and HPV viral load, concluding that a higher HPV16/18 load was more closely associated with CST IV in women younger than 50 years old. Thus, the improvement in the VMB structure following cervical resection may be associated with the reduction in viral load resulting from the surgery.

Persistent HPV infection following surgery is associated with vaginal microbiome dysbiosis. This dysbiosis is linked not only to anaerobic bacteria associated with BV, but also to pathogens associated with aerobic vaginitis (AV), as identified by wet mount microscopy, including Enterococcus, Escherichia coli, Staphylococcus, and Streptococcus. In the differential analysis of the VMB, the relative abundance of Escherichia-Shigella at the genus level was significantly higher in the postoperative HPV-positive group compared to the postoperative HPV-negative group (2.6% vs 0.1%, *P* = 0.01). Ma et al. (2024) investigated the VMB of 207 patients with cervical cancer and cervical intraepithelial neoplasia post-surgery and discovered that microbiome dysbiosis was associated with persistent HPV infection and lesion recurrence. The primary characteristics of this dysbiosis were an elevated pH (>4.5) and the occurrence of AV, although the specific pathogens associated with AV were not specified. A recent study (Grincevičienė et al., 2024) indicated that AV is more prevalent in HSIL and cervical cancer than in low-grade squamous intraepithelial lesion(LSIL), whereas BV may more frequently represent HPV infection or LSIL. The persistent overgrowth of AV-associated pathogens in the VMB may suggest a poor prognosis, and thus, further research is warranted.

Scardovia was the other differential genus between postoperative HPV-positive group and postoperative HPV-negative group (0.8% vs<0.1%, *P* = 0.01). It belongs to the Bifidobacteriaceae family, with most research focusing on its association with dental caries in the oral cavity (Kameda et al., 2020; Spatafora et al., 2024). There have been reports on its enrichment in urinary tract infections (Mekadim et al., 2019) and in the VMB of healthy pregnant women (Xiao et al., 2023), but no studies similar to ours have been conducted.

Van de Wijgert JHHM’s team (van de Wijgert et al., 2020) classified vaginal flora into three major categories: dominant lactobacillus, commonly present BV-associated anaerobes, and less prevalent but more pathogenic bacteria, the latter including Streptococcus, Staphylococcus, Enterococcus, Escherichia-Shigella, and so on. Streptococcus is a common pathogen associated with AV. Our study observed high relative abundances across all three groups (P1 vs P2 vs N, 4.8% vs 3.2% vs 3.4%), with no significant differences among them (*P* > 0.05). Although our research process was very rigorous, it leads us to consider whether this phenomenon is related to errors during the collection or storage of the samples. Perhaps similar studies of this region in the future can provide clues to the answer.

In the correlation analysis of vaginal microbes, no genus was found to be positively related to Lactobacillus, but Gardnerella, Atopobium, Prevotella, Sneathia, Bifidobacterium and Streptococcus were all negatively related to it. This is easy to understand. Lactobacillus plays a protective role in the vaginal microenvironment by producing lactic acid, hydrogen peroxide, bacteriocins and biosurfactants, and through competitive exclusion, to promote a healthy VMB and establish a defense against pathogens(Chee et al., 2020; So et al., 2020; Chen et al., 2021). When the level of Lactobacillus decreases, those pathogens will proliferate.

Gardnerella, the most commonly identified microorganism in BV, has been found to be positively correlated with Atopobium. The relationship between these two bacteria is well-known for contributing to the formation of polymicrobial biofilms associated with BV. Gardnerella plays a major role in the formation of biofilms, and Atopobium attaches to Gardnerella within the biofilm (Muzny et al., 2020). This is also the reason why Atopobium is rarely detected in the absence of Gardnerella (Sehgal et al., 2021). Moreover, the presence of Atopobium or other anaerobes associated with BV increase the harmfulness of Gardnerella. Besides that, Gardnerella was also found to be negatively associated with pathogens such as Klebsiella, Bifidobacterium, Streptococcus, Escherichia-Shigella, and Anaerococcus in cases of HPV infection. Vaneechoutte et al. (2019) conducted whole genome sequence analysis on 81 Gardnerella strains and identified the existence of at least 13 subgroups. Different genomic properties may present different pathological features and determine pathogenicity or not. Further research is needed regarding the relationship between Gardnerella and other bacteria, particularly in the context of HPV infection.

Bifidobacterium and Streptococcus exhibited a strong positive correlation across all three groups. As is well known, Bifidobacterium is a widely distributed commensal bacterial genus in the gut microbiome that exhibits beneficial pro-homeostatic and anti-inflammatory immunomodulatory properties (Gavzy et al., 2023). But in the vaginal environment, descriptions of Bifidobacteria are common in the Lactobacillus-deficient vaginal communities. France et al. (2020) improved the classification of VMB based on big data and new algorithms, dividing it into nine types CST, and further classified CST IV-C into five subtypes. Among the subtypes, Bifidobacterium was associated with a lower vaginal pH, whereas the majority of Streptococcus communities typically exhibited a higher pH. CST IV-C1 (Streptococcus-dominated) and CST IV-C3 (Bifidobacterium-dominated) were common among women of reproductive age. However, the synergistic relationship between them remains unclear.

The results of the phenotypic analysis for VMB were consistent with those of the microbial structure analysis. The relative abundances of the Anaerobic and Forms Biofilms phenotypes were significantly higher in the HPV-positive groups compared to the HPV-negative group (*P* < 0.05), with Group P2 exhibiting the highest levels. This may intuitively explain some of the reasons for the postoperative persistent HPV infection, such as overgrowth of anaerobic bacteria and the harmful effects of related biofilms. On the other hand, due to the highest abundance of Lactobacillus, the postoperative persistent HPV-negative group exhibited the best performance in term of Facultatively Anaerobic phenotype.

The limitations of this study should be clarified. Firstly, being a cross-sectional study, we cannot dynamically observe the changes in the microbiome of the same individual before and after cervical conization, which may influence the research results due to individual differences. Secondly, we have identified certain statistically significant relationships between some bacteria; however, due to the limitations of current basic research, we cannot adequately explain their intrinsic connections. Thirdly, we did not complete further genome sequencing, which limited our analysis to the species level.

## 5 Conclusions

The vaginal microbiota of women with persistent HPV infection following cervical conization is characterized by the coexistence of Lactobacillus dominance and increased microbial diversity. Anaerobic bacteria and biofilm formation may play a significant role in the persistence of HPV infection post-surgery. The role of Gardnerella in vaginal flora under an HPV-infected state warrants further study.

## Data Availability

The datasets presented in this study can be found in online repositories. The names of the repository/repositories and accession number(s) can be found in the article/supplementary material.

## References

[B1] Bai B. Tuerxun G. Tuerdi A. Maimaiti R. Sun Y. Abudukerimu A. (2024). Analysis of vaginal flora diversity and study on the role of Porphyromonas asaccharolytica in promoting IL-1β in regulating cervical cancer. Sci. Rep. 14, 21731. doi: 10.1038/s41598-024-73146-9 39289490 PMC11408518

[B2] Borgdorff H. Gautam R. Armstrong S. D. Xia D. Ndayisaba G. F. van Teijlingen N. H.. (2015). Cervicovaginal microbiome dysbiosis is associated with proteome changes related to alterations of the cervicovaginal mucosal barrier. Mucosal Immunol. 9, 621–633. doi: 10.1038/mi.2015.86 26349657

[B3] Borgogna J. C. Shardell M. D. Santori E. K. Nelson T. M. Rath J. M. Glover E. D.. (2020). The vaginal metabolome and microbiota of cervical HPV-positive and HPV-negative women: a cross-sectional analysis. BJOG 127, 182–192. doi: 10.1111/1471-0528.15981 31749298 PMC6982399

[B4] Caselli E. D’Accolti M. Santi E. Soffritti I. Conzadori S. Mazzacane S.. (2020). Vaginal microbiota and cytokine microenvironment in HPV clearance/persistence in women surgically treated for cervical intraepithelial neoplasia: an observational prospective study. Front. Cell Infect. Microbiol. 10. doi: 10.3389/fcimb.2020.540900 PMC7676899 33251154

[B5] Castle P. E. Fetterman B. Akhtar I. Husain M. Gold M. A. Guido R.. (2009). Age-appropriate use of human papillomavirus vaccines in the U.S. Gynecol. Oncol. 114, 365–369. doi: 10.1016/j.ygyno.2009.04.035 19464729 PMC2729751

[B6] Chee W. J. Y. Chew S. Y. Than L. T. L. (2020). Vaginal microbiota and the potential of Lactobacillus derivatives in maintaining vaginal health. Microb. Cell Fact. 19, 203. doi: 10.1186/s12934-020-01464-4 33160356 PMC7648308

[B7] Chen X. Lu Y. Chen T. Li R. (2021). The female vaginal microbiome in health and bacterial vaginosis. Front. Cell Infect. Microbiol. 11. doi: 10.3389/fcimb.2021.631972 PMC8058480 33898328

[B8] Chen Y. Qiu X. Wang W. Li D. Wu A. Hong Z.. (2020). Human papillomavirus infection and cervical intraepithelial neoplasia progression are associated with increased vaginal microbiome diversity in a Chinese cohort. BMC Infect. Dis. 20, 629. doi: 10.1186/s12879-020-05324-9 32842982 PMC7449047

[B9] D’Augè T. G. Di Donato V. Giannini A. (2024). Strategic approaches in management of early-stage cervical cancer: A comprehensive editorial. Clin. Exp. Obstet. Gynecol. 51, 235. doi: 10.31083/j.ceog5110235

[B10] Fang B. Li Q. Wan Z. OuYang Z. Zhang Q. (2022). Exploring the association between cervical microbiota and HR-HPV infection based on 16S rRNA gene and metagenomic sequencing. Front. Cell Infect. Microbiol. 12. doi: 10.3389/fcimb.2022.922554 PMC9253761 35800388

[B11] France M. T. Ma B. Gajer P. Brown S. Humphrys M. S. Holm J. B.. (2020). VALENCIA: a nearest centroid classification method for vaginal microbial communities based on composition. Microbiome 8, 166. doi: 10.1186/s40168-020-00934-6 33228810 PMC7684964

[B12] Gao W. Weng J. Gao Y. Chen X. (2013). Comparison of the vaginal microbiota diversity of women with and without human papillomavirus infection: a cross-sectional study. BMC Infect. Dis. 13, 271. doi: 10.1186/1471-2334-13-271 23758857 PMC3684509

[B13] Gavzy S. J. Kensiski A. Lee Z. L. Mongodin E. F. Ma B. Bromberg J. S. (2023). Bifidobacterium mechanisms of immune modulation and tolerance. Gut Microbes 15, 2291164. doi: 10.1080/19490976.2023.2291164 38055306 PMC10730214

[B14] Grincevičienė Š. Vaitkienė D. Kanopienė D. Vansevičiūtė Petkevičienė R. Sukovas A. Celiešiūtė J.. (2024). Aerobic vaginitis is associated with carbonic anhydrase IX in cervical intraepithelial neoplasia. Sci. Rep. 14, 8789. doi: 10.1038/s41598-024-57427-x 38627429 PMC11021548

[B15] Huang R. Liu Z. Sun T. Zhu L. (2024). Cervicovaginal microbiome, high-risk HPV infection and cervical cancer: Mechanisms and therapeutic potential. Microbiol. Res. 287, 127857. doi: 10.1016/j.micres.2024.127857 39121703

[B16] Kameda M. Abiko Y. Washio J. Tanner A. C. R. Kressirer C. A. Mizoguchi I.. (2020). Sugar metabolism of scardovia wiggsiae, a novel caries-associated bacterium. Front. Microbiol. 11. doi: 10.3389/fmicb.2020.00479 PMC7109253 32269556

[B17] Li M. Zhao C. Zhao Y. Li J. Wei L. (2022). Age-stratified analysis of vaginal microbiota dysbiosis and the relationship with HPV viral load in HPV-positive women. J. Immunol. Res. 2022, 1372926. doi: 10.1155/2022/1372926 35935589 PMC9348945

[B18] Liu Y. Zhao X. Wu F. Chen J. Luo J. Wu C.. (2024). Effectiveness of vaginal probiotics Lactobacillus crispatus chen-01 in women with high-risk HPV infection: a prospective controlled pilot study. Aging (Albany NY) 16, 11446–11459. doi: 10.18632/aging.206032 39058300 PMC11315381

[B19] Ma Y. Wan L. Li R. Chen X. Wang H. (2024). Impact of postsurgical vaginal microbiome on high-risk HPV infection and recurrence risk in patients with cervical cancer and intraepithelial neoplasia: A retrospective study. Gynecol. Oncol. Rep. 55, 101506. doi: 10.1016/j.gore.2024.101506 39308899 PMC11416653

[B20] Mekadim C. Bunešová V. Vlková E. Hroncová Z. Killer J. (2019). Genetic marker-based multi-locus sequence analysis for classification, genotyping, and phylogenetics of the family Bifidobacteriaceae as an alternative approach to phylogenomics. Antonie Van Leeuwenhoek. 112, 1785–1800. doi: 10.1007/s10482-019-01307-2 31368048

[B21] Muzny C. A. Łaniewski P. Schwebke J. R. Herbst-Kralovetz M. M. (2020). Host-vaginal microbiota interactions in the pathogenesis of bacterial vaginosis. Curr. Opin. Infect. Dis. 33, 59–65. doi: 10.1097/QCO.0000000000000620 31789672 PMC7265982

[B22] Palma E. Recine N. Domenici L. Giorgini M. Pierangeli A. Panici P. B. (2018). Long-term Lactobacillus rhamnosus BMX 54 application to restore a balanced vaginal ecosystem: a promising solution against HPV-infection. BMC Infect. Dis. 18, 13. doi: 10.1186/s12879-017-2938-z 29304768 PMC5756375

[B23] Ravel J. Gajer P. Abdo Z. Schneider G. M. Koenig S. S. McCulle S. L.. (2011). Vaginal microbiome of reproductive-age women. Proc. Natl. Acad. Sci. U S A. 108 Suppl 1, 4680–4687. doi: 10.1073/pnas.1002611107 20534435 PMC3063603

[B24] Sehgal P. G. Dadwal R. Sharma B. Sehgal A. Bagga R. Chopra S.. (2021). Detection of co-infection of Gardnerella vaginalis and Atopobium vaginae using qualitative PCR: A better predictor of bacterial vaginosis. Anaerobe 69, 102343. doi: 10.1016/j.anaerobe.2021.102343 33582302

[B25] Sharifian K. Shoja Z. Jalilvand S. (2023). The interplay between human papillomavirus and vaginal microbiota in cervical cancer development. Virol. J. 20, 73. doi: 10.1186/s12985-023-02037-8 37076931 PMC10114331

[B26] So K. A. Yang E. J. Kim N. R. Hong S. R. Lee J. H. Hwang C. S.. (2020). Changes of vaginal microbiota during cervical carcinogenesis in women with human papillomavirus infection. PloS One 15, e0238705. doi: 10.1371/journal.pone.0238705 32941440 PMC7498004

[B27] Spatafora G. Li Y. He X. Cowan A. Tanner A. C. R. (2024). The evolving microbiome of dental caries. Microorganisms 12, 121. doi: 10.3390/microorganisms12010121 38257948 PMC10819217

[B28] Uren A. Fallen S. Yuan H. Usubütün A. Küçükali T. Schlegel R.. (2005). Activation of the canonical Wnt pathway during genital keratinocyte transformation: a model for cervical cancer progression. Cancer Res. 65, 6199–6206. doi: 10.1158/0008-5472.CAN-05-0455 16024621

[B29] van der Heijden E. Lopes A. D. Bryant A. Bekkers R. Galaal K. (2015). Follow-up strategies after treatment (large loop excision of the transformation zone (LLETZ)) for cervical intraepithelial neoplasia (CIN): Impact of human papillomavirus (HPV) test. Cochrane Database Syst. Rev. 1, CD010757. doi: 10.1002/14651858.CD010757 25562623 PMC6457759

[B30] van de Wijgert J. H. H. M. Verwijs M. C. Gill A. C. Borgdorff H. van der Veer C. Mayaud P. (2020). Pathobionts in the vaginal microbiota: individual participant data meta- analysis of three sequencing studies. Front. Cell Infect. Microbiol. 10. doi: 10.3389/fcimb.2020.00129 PMC7174631 32351902

[B31] Vaneechoutte M. Guschin A. Van Simaey L. Gansemans Y. Van Nieuwerburgh F. Cools P. (2019). Emended description of Gardnerella vaginalis and description of Gardnerella leopoldii sp. nov., Gardnerella piotii sp. nov. and Gardnerella swidsinskii sp. nov., with delineation of 13 genomic species within the genus Gardnerella. Int. J. Syst. Evol. Microbiol. 69, 679–687. doi: 10.1099/ijsem.0.003200 30648938

[B32] Wu S. Ding X. Kong Y. Acharya S. Wu H. Huang C.. (2021). The feature of cervical microbiota associated with the progression of cervical cancer among reproductive females. Gynecol. Oncol. 163, 348–357. doi: 10.1016/j.ygyno.2021.08.016 34503848

[B33] Xia Y. Feng Y. Qin T. Zhao X. Lu J. Ma C. (2022). Characteristics of vaginal microbiome in reproductive-age females with HPV infection in Xinjiang, China. Evid. Based Complement Alternat. Med., 7332628. doi: 10.1155/2022/7332628 36387363 PMC9643059

[B34] Xiao Y. Huang S. Yu W. Ni Y. Lu D. Wu Q.. (2023). Effects of emergency/nonemergency cervical cerclage on the vaginal microbiome of pregnant women with cervical incompetence. Front. Cell Infect. Microbiol. 13. doi: 10.3389/fcimb.2023.1072960 PMC10034410 36968117

[B35] Zeng M. Li X. Jiao X. Cai X. Yao F. Xu S.. (2023). Roles of vaginal flora in human papillomavirus infection, virus persistence and clearance. Front. Cell Infect. Microbiol. 12. doi: 10.3389/fcimb.2022.1036869 PMC9848591 36683675

